# SMAC mimetic Debio 1143 synergizes with taxanes, topoisomerase inhibitors and bromodomain inhibitors to impede growth of lung adenocarcinoma cells

**DOI:** 10.18632/oncotarget.6138

**Published:** 2015-10-16

**Authors:** Casey G. Langdon, Norbert Wiedemann, Matthew A. Held, Ramanaiah Mamillapalli, Pinar Iyidogan, Nicholas Theodosakis, James T. Platt, Frederic Levy, Gregoire Vuagniaux, Shaomeng Wang, Marcus W. Bosenberg, David F. Stern

**Affiliations:** ^1^ Department of Pathology and Yale Cancer Center, Yale University School of Medicine, New Haven, CT, USA; ^2^ Debiopharm International SA, Lausanne, Switzerland; ^3^ Breast Medical Oncology Group, Yale Cancer Center, New Haven, CT, USA; ^4^ Departments of Dermatology and Yale Cancer Center, Yale University School of Medicine, New Haven, CT, USA; ^5^ Comprehensive Cancer Center, University of Michigan, Ann Arbor, MI, USA; ^6^ Massachusetts General Hospital Cancer Center, Harvard Medical School, Charlestown, MA, USA

**Keywords:** SMAC mimetic, combination therapy, lung adenocarcinoma, high throughput screening, bromodomain inhibitor

## Abstract

Targeting anti-apoptotic proteins can sensitize tumor cells to conventional chemotherapies or other targeted agents. Antagonizing the Inhibitor of Apoptosis Proteins (IAPs) with mimetics of the pro-apoptotic protein SMAC is one such approach. We used sensitization compound screening to uncover possible agents with the potential to further sensitize lung adenocarcinoma cells to the SMAC mimetic Debio 1143. Several compounds in combination with Debio 1143, including taxanes, topoisomerase inhibitors, and bromodomain inhibitors, super-additively inhibited growth and clonogenicity of lung adenocarcinoma cells. Co-treatment with Debio 1143 and the bromodomain inhibitor JQ1 suppresses the expression of c-IAP1, c-IAP2, and XIAP. Non-canonical NF-κB signaling is also activated following Debio 1143 treatment, and Debio 1143 induces the formation of the ripoptosome in Debio 1143-sensitive cell lines. Sensitivity to Debio 1143 and JQ1 co-treatment was associated with baseline caspase-8 expression. *In vivo* treatment of lung adenocarcinoma xenografts with Debio 1143 in combination with JQ1 or docetaxel reduced tumor volume more than either single agent alone. As Debio 1143-containing combinations effectively inhibited both *in vitro* and *in vivo* growth of lung adenocarcinoma cells, these data provide a rationale for Debio 1143 combinations currently being evaluated in ongoing clinical trials and suggest potential utility of other combinations identified here.

## INTRODUCTION

The ability of tumor cells to evade apoptosis is crucial for tumor biology. The inhibitor of apoptosis proteins (IAPs) are important mediators of the anti-apoptotic phenotype. IAPs are highly expressed in most tumors [1]. All eight IAP family members have at least one BIR (baculovirus IAP repeat) domain that interacts with and inhibits the catalytic activity of caspases [2]. Three IAP family members - cIAP1, cIAP2, and XIAP - also have RING (really interesting new gene) domains with E3 ubiquitin ligase activity [2]. These three IAPs modulate ubiquitin-mediated proteasomal degradation of proteins and ubiquitin-mediated signaling events [2]. IAP-mediated non-degradative ubiquitination of RIP1 leads to activation of the inhibitor of NF-κB kinase (IKK), phosphorylation and proteasomal degradation of IκBα, and nuclear localization of the transcriptional effector subunits of NF-κB [2–4]. IAP-mediated ubiquitination of NIK (NF-κB-inducing kinase) inactivates the non-canonical NF-κB pathway [2, 5, 6].

Second mitochondrial-derived activator of caspases (SMAC) is an endogenous inhibitor of IAPs [7]. Stimulation of apoptotic pathways releases SMAC from mitochondria; the amino-terminus of SMAC is then proteolytically cleaved, exposing a four amino acid motif that binds to BIR domains in XIAP, cIAP1, and cIAP-2. This prevents IAPs from binding caspases [7–10]. SMAC also promotes the auto-ubiquitination and subsequent degradation of cIAP1 and cIAP2, but not XIAP [7, 11].

Small molecule SMAC mimetics are in various stages of clinical development. They function similarly to endogenous SMAC by antagonizing the inhibition of caspases mediated by XIAP and by inducing the auto-ubiquitination of cIAP1 or cIAP2; subsequent NF-κB-induced expression of TNFα further activates pro-apoptotic TNFR1 (TNFα receptor) signaling [12–14]. Downregulation of the anti-apoptotic protein c-FLIP is necessary for sensitivity of breast cancer cell lines to SMAC mimetics [15]. SMAC mimetic-induced loss of IAP function is also associated with formation of the cell-death inducing complex - the ripoptosome [16, 17]. However, ripoptosome formation does not require TNFα expression. The ripoptosome mediates both caspase-8-dependent apoptosis and caspase-independent necroptosis [16].

Only a subset of non-small cell lung cancer cell lines responds to SMAC mimetics as single agents, supporting the need for combination therapy treatment [14]. SMAC mimetics synergize with conventional chemotherapeutics including paclitaxel, gemcitabine, cisplatin, carboplatin [18] and the topoisomerase inhibitor SN-38 in a variety of tumors (reviewed in [2]). SMAC mimetics also sensitize tumor cells to radiation therapy [19, 20]. Here, we describe a sensitization screen using the SMAC mimetic Debio 1143 (AT-406) to identify effective partner agents for the treatment of lung adenocarcinoma cells with various driver and resistance mutations. Debio 1143 is an orally available IAP inhibitor being developed in combination with chemo and radiotherapy in various solid tumors (ClinicalTrials.gov Identifier: NCT01930292) [21]. The combination screen revealed novel combinations with Debio 1143 that induce apoptosis, intercellular signaling alterations, and xenograft efficacy.

## RESULTS

### Lung adenocarcinoma cell lines are differentially responsive to Debio 1143

Six lung adenocarcinoma cell lines, with *KRAS*, *EGFR*, or *ALK* driver mutations were evaluated for Debio 1143 dose-dependent growth inhibition to identify optimal concentrations for use in combination assays (Table 1). The two most sensitive cell lines, H1975 and H820, have EGFR driver mutations and are resistant to erlotinib as they harbor gatekeeper T790M mutations. The T790M substitution does not affect sensitivity to Debio 1143, as matched pairs of erlotinib-sensitive parental and derivative erlotinib-resistant (T790M) cell lines have similar Debio 1143 dose response profiles (Figure S1A). Two of the other four lines tested - H2030 (*KRAS* mutation) and H2228 (*EML4*-*ALK* translocation) - were less sensitive to Debio 1143. A549 and H1650 cells were resistant (Table 1). Hence, subsets of lung adenocarcinoma lines with three different common driver mutations are sensitive to Debio 1143.

**Table 1:** GI_50_, GI_25_, and GI_10_ values for Debio 1143 in the panel of lung adenocarcinoma cell lines used for sensitization screening

Cell line	Mutation Status	Debio1143		
		*GI50*	*GI25*	*GI10*
A549	KRAS (G12S), STK11, SMARCA4, CDKN2A	100*	50.35	18.02
H2030	KRAS (G12C), STK11, SMARCA4, TP53	65.39	27.35	7.15
H1650	EGFR (746-750del), TP53, CDKN2A, PI3KCA	100*	73.23	14.94
H1975	EGFR (L858R, T790M), TP53, CDKN2A, PI3KCA	21.30	2.29	0.28
H820	EGFR (746-750del, T790M), MET amp	2.55	0.65	0.25
H2228	EML4-ALK variant 3, CDKN2A, RB1, TP53	38.87	5.36	0.40

* GI50 never achieved. Highest concentration used in screening experiments

**Table 2:** Top 27 agents in all cell lines, or either mutant *EGFR* or *KRAS* lung adenocarcinoma cells, that best sensitized cells to Debio 1143 according to AUC metric

143 all cell lines top 27		1143 EGFR		1143 KRAS	
Compound	Target	Compound	Target	Compound	Target
*Paclitaxel*	microtubule	*Paclitaxel*	microtubule	Flavopiridol	CDKs
Triptolide	NFkB, CIAP	Homoharringtonine	protein synthesis	BI-2536	PLK
JK 184	GLI, microtubules	JK 184	GLI, microtubules	*Mitomycin* C	microtubule
BI-2536	PLK	*Vinorelbine*	microtubule	Obatoclax	BCL2
*Venorelbine*	Microtubule	*Topotecan*	topoisomerase	JK 184	GLI, microtubules
*Topotecan*	topoisomerase	Docetaxel	microtubule	*Paclitaxel*	microtubule
*Docetaxel*	microtubule	KP372-1	PDK1/AKT/FLT	Temsirolimus*	mTOR
KP372-1	PDK1/AKT/FLT	*SN-38*	topoisomerase	Vorinostat*	HDAC
Flavopiridol	CDKs	Triptolide	NFkB, CIAP	*Bortezomib*	proteasome
*SN-38*	topoisomerase	*Gemcitabine*	nucleoside analog	Rapamycin*	*mTOR*
*Bortezomib*	proteasome	BI-2536	PLK	Triptolide	NFkB, CIAP
*Gemcitabine*	nucleoside analog	Foretinib	MET/AXL/VEGFR	Vinorelbine*	microtubule
*Homoharringtonine*	protein synth	17-DMAG	HSP90	MS-275	HDAC1, 3
*Dasatinib*	Abl, Src	GDC-0941	PI3K	SB 218078	Chk1
*Dactinomycin*	transcription	MLN-8237	Aurora	CIP 13-74	MEK
GDC-0941	PI3K	ABT-263	Bc1-2 family	Triapine	ribonucleotide reductase
MS-275	HDAC 1,3	*Dasatinib*	Abl, Src	Homoharringtonine	protein synth
IMD 0354	IKK-2	IMD 0354	IKK2	JQ1	bromodomain
AZD-7762	CHK 1/2	Flavopiridol	CDKs	*Vandetanib*	EGFR/VEGFR/RET
Foretinib	MET/AXL/VEFGR	MK-1775	WEE1	STA-4783*	ROS, pro-apoptosis
MLN-8237	Aurora	Dactinomycin*	transcription	*Topotecan*	topoisomerase
NVP-TAE684	ALK	BIBW-2992	EGFr/ERBB2	*AZD-7762*	CHK 1/2
ABT-263	BCL2 family	Bortezomib*	proteasome	KP372-1	PDK1/AKT/FLT
MK-1775	WEE1	*Digoxin*	NaK ATPase	BEZ-235	P13K/mTOR
JQ1	bromodomain	PF 431396	FAK/PYK2	*Docetaxel*	microtubule
BIBW-2992	EGFR/ERBB2	HBX-41108	USP7	Tozesertib	Aurora
PF 431396	FAK.PYK2	*Carfilzomib*	proteasome	*GSK1120212*	MEK Trametinib

Compounds marked in green and italicized are FDA-approved

* Indicates that compound growth response curves are not as good as ranking indicates

### Debio 1143 combination screen

**Figure 1:**
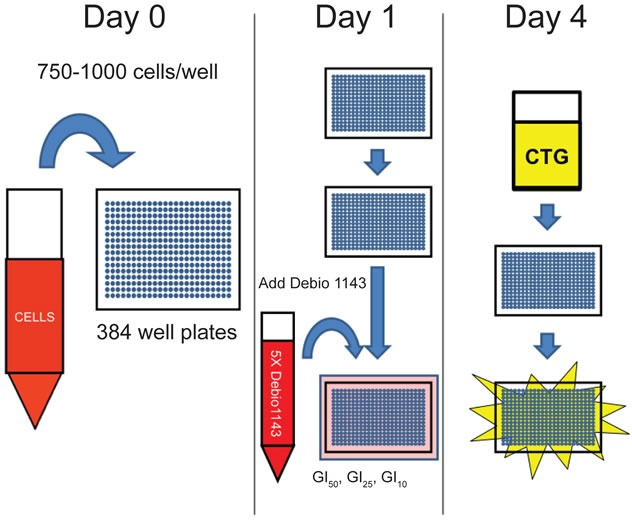
Schematic of Debio 1143 sensitization screen

**Figure 2:**
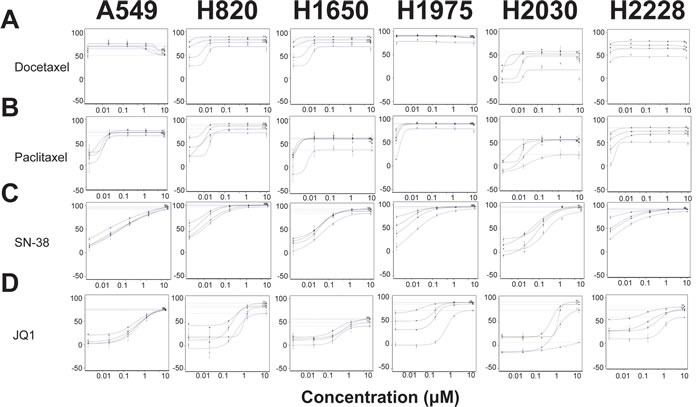
Taxanes, topoisomerase inhibitors, and bromodomain inhibitors sensitize lung adenocarcinoma cell lines to Debio 1143 treatment Growth inhibition curves of docetaxel **A.**, paclitaxel **B.**, SN-38 **C.**, and JQ1 **D.** in combination with Debio 1143. Blue curves indicate growth inhibitory response to docetaxel, paclitaxel, SN-38, or JQ1 in each respective cell line. The three black curves indicate the response to the combination of each respective agent and Debio 1143 in each cell line. Growth inhibition is plotted along the y-axis of each graph. Debio 1143 concentrations used for each cell line are found in Table 1.

We sought to identify partner agents that would augment efficacy and potency of Debio 1143, using a sensitization combination screening format illustrated in Figure 1. The six cell lines described in Table 1 were screened against 128 agents (Table S1). These compounds inhibit a range of different target classes including components of growth factor signaling pathways, metabolic and epigenetic modulators, and conventional chemotherapeutics. An Area Under the Curve (AUC) metric quantified the impact of combinations that inhibited growth more effectively and/or more potently than either single agent. The top 20% of partner agents ranked by AUC (Table S1) include several candidates that are in clinical trials or are already US FDA-approved (Table 2). Microtubule stabilizers, such as docetaxel (Figure 2A) and paclitaxel (Figure 2B) were some of the highest scoring compounds across the panel of six cell lines screened in combination with Debio 1143. Topoisomerase inhibitors, such as SN-38 (Figure 2C) or topotecan (Figure S2A), also scored highly using the AUC metric. Broadly acting small molecules, such as triptolide, JK184, gemcitabine, MS-275 (HDAC inhibitor), and bortezomib (data not shown) also gave robust additive responses in the sensitization screen. A subset of lung adenocarcinoma cell lines was also sensitive to bromodomain inhibition with both iBET (Figure S2B) and JQ1 (Figure 2D).

Three of the cell lines have EGFR driver mutations but are resistant to EGFR inhibition through the common T790M resistance mutation (H1975, and H820, which is also *MET*-amplified,) or other mechanisms (H1650). These cell lines were more sensitive to several receptor tyrosine kinase inhibitors, including the broad kinase inhibitors dasatinib and foretinib. The third-generation EGFR inhibitor, afatinib (BIBW-2992), combined super-additively with Debio 1143 to inhibit growth of mutant *EGFR* lung adenocarcinomas. Debio 1143 sensitized cells to co-treatment with a second agent for one of the two mutant *KRAS* cell lines in the screen - H2030. This is especially interesting as H2030 is relatively resistant to approximately 100 single agents that we tested with a broad range of targets (data not shown). Debio 1143 sensitized H2030 cells to inhibition of Polo-like kinase, PI3 kinase, MEK, and BCL-2 family members (data not shown). Other combinations with Debio 1143 were no more effective than either agent alone, or were antagonistic. They included receptor tyrosine kinase inhibitors, such as AZD-4547, sunitinib, and crizotinib. AKT inhibition also did not sensitize cells to Debio 1143 treatment. Taken together, the screen revealed several combinations with enhanced growth inhibitory activity on a variety of lung adenocarcinoma cell lines, as well as several combinations that did not enhance growth inhibition.

### Synergistic Debio 1143 combinations

**Figure 3:**
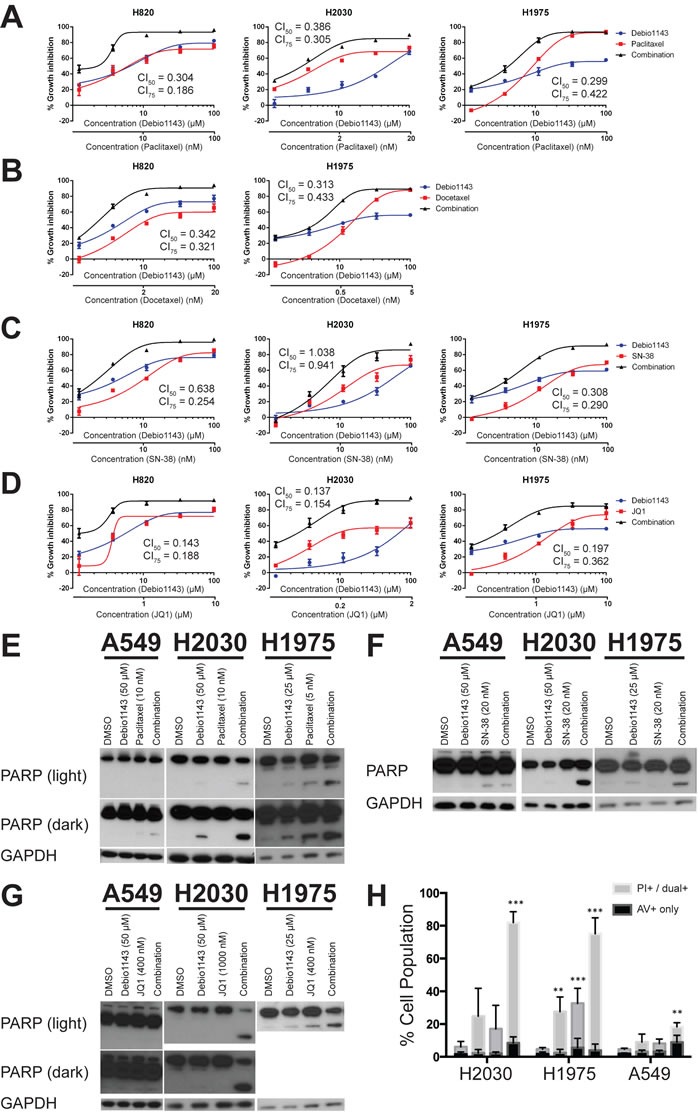
Debio 1143 synergizes with several agents to inhibit growth of lung adenocarcinoma cell lines and induce apoptosis **A.**-**D.** Fixed-concentration growth inhibition assays were performed with four different Debio 1143-containing combinations - **A.** Debio 1143 and paclitaxel; **B.** Debio 1143 and docetaxel; **C.** Debio 1143 and SN-38; **D.** Debio 1143 and JQ1. (E-G) Immunoblots with indicated primary antibodies following treatment with Debio 1143 and paclitaxel **E.**, SN-38 **F.**, or JQ1 **G.** combinations. **H.** Annexin V/propidium iodide flow cytometry following Debio 1143 and/or JQ1 treatment. Annexin V (AV+) only stained cells considered early apoptotic. Propidium iodide-only stained plus dual Annexin V- and propidium iodide-stained positive cells considered late apoptotic. Both early and late apoptotic populations added together for statistical purposes. ** *p* < 0.01, *** *p* < 0.001. H1975 = Debio 1143 (25 μM), JQ1 (400 nM); H2030 = Debio 1143 (50 μM), JQ1 (1 μM); A549 = Debio 1143 (50 μM), JQ1 (400 nM).

Synergistic growth inhibition could not be determined with the small number of dose points used initially, but can be formally evaluated by calculating combination index values [26]. We were particularly interested in the taxanes as they were among the highest scoring combinations by the AUC metric, and because the combination of Debio 1143 with paclitaxel and carboplatin is in clinical trials for squamous non-small cell lung cancer, platinum-refractory ovarian cancer, and triple-negative breast cancer (NCT01930292). Debio 1143 was more effective and potent at inhibiting growth in combination with either paclitaxel (Figure 3A) or docetaxel (Figure 3B). Debio 1143 was also more effective in combination with SN-38 (the active metabolite of irinotecan) or with the bromodomain inhibitor JQ1 (Figure 3C and 3D, respectively). Almost all combinations tested in Figure 2 were synergistic based upon the Chou-Talalay combination indices derived from the curves in Figure 3A-3D. The only exception was the combination of Debio 1143 and SN-38 in H2030 cells; this interaction was additive, but not synergistic.

We next evaluated the effects of these three combinations on cell clonogenicity. Debio 1143 reduced clonogenicity of H1975 and H2030 cells, but not A549 cells, consistent with the original screening data (Figure S3). Debio 1143 in combination with all three agents substantially reduced clonogenicity compared to vehicle-treated cells. This included A549 cells, which were resistant to Debio 1143 as a single agent (Figure S3).

Debio 1143, alone and in combinations, could affect cell accumulation and clonogenicity through impact on cell proliferation and/or on cell survival. Over a period of 24 hours, Debio 1143 alone induced moderate poly-ADP ribose polymerase (PARP) cleavage in H1975 and H2030 cells; cleaved PARP levels were greatly enhanced in the presence of paclitaxel, SN38, or JQ1 in H1975 and H2030 cells (Figure 3E, 3F, and 3G). Debio 1143 induced no PARP cleavage in A549 cells, which are resistant to this agent in CellTiterGlo and clonogenic growth assays. In A549 cells, the extent of PARP cleavage in combinations was similar to that induced by the single partner agents. The impact of longer-term 72 hours treatment on apoptosis was determined by Annexin V/propidium iodide flow cytometry. Debio 1143 plus JQ1 induced apoptosis in the combination-sensitive lines H1975 and H2030, but not the combination-resistant line A549 (Figure 3H). Overall, Debio 1143 is effective as a single agent and induces apoptosis in combination with compounds with diverse mechanisms of action.

### Signaling impact of Debio 1143 and JQ1 co-treatment

Modulation of PI3K and MAPK signaling has shown benefit in lung adenocarcinoma cell lines. Co-treatment with Debio 1143 and JQ1 reduced pAKT(S473) and p70S6K (phospho and total) in H2030 and A549 cells, but not in H1975 (Figure S4). The Debio 1143/JQ1 combination reduced pERK in A549 and H2030, but increased pERK in H1975 cells (Figure S4). Hence, sensitivity to Debio 1143 plus JQ1 was not tightly correlated with PI3K or MAPK signaling in these lines.

**Figure 4:**
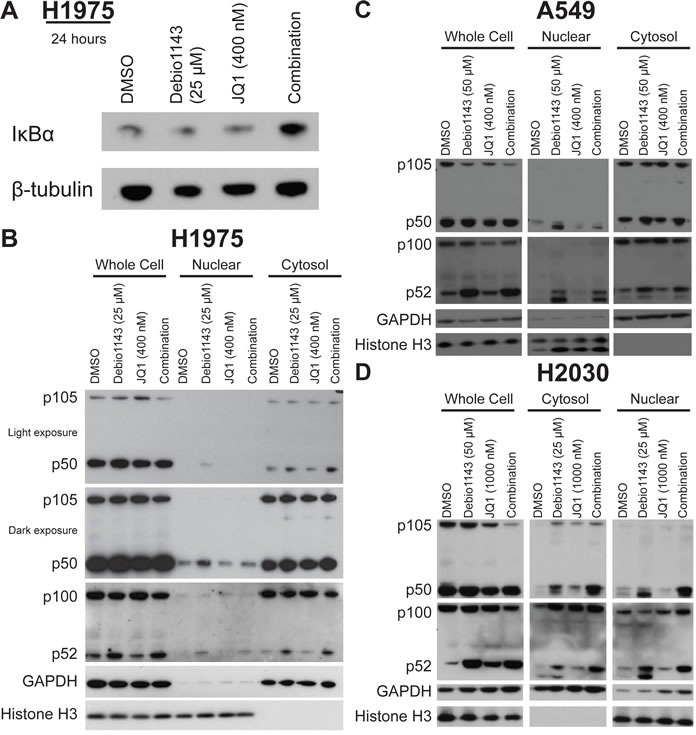
Debio 1143 and JQ1 treatment activates NF-κB signaling **A.** Immunoblots of IκBα protein levels in H1975 cells after 24 hour treatment. Cellular fractionation of **B.** H1975, **C.** A549, and **D.** H2030 cells following 24 hour treatment with Debio 1143 and/or JQ1. Immunoblots with canonical and non-canonical NF-κB signaling components.

SMAC mimetics and bromodomain inhibitors modulate the NF-κB pathway [5, 6, 12]. We evaluated the impact of Debio 1143 and JQ1, alone or in combination, on the canonical and non-canonical arms of the NF-κB pathway. The combination, but neither single agent, elevated IκBα, an NF-κB inhibitor (Figure 4A). Activity of the non-canonical and canonical NF-κB pathway is marked by nuclear localization of effectors p52 and p50, respectively. Debio 1143, but not JQ1, induced nuclear localization of p52 in each cell line tested - H1975, A549, and H2030, with no augmentation by the combination (Figure 4B, 4C, and 4D). Debio 1143 induced nuclear localization of p50 in all three cell lines (Figure 4B, 4C, and 4D). However, the combination restored nuclear p50 protein levels to baseline levels in H1975 cells (Figure 4B). The combination only slightly decreased nuclear p50 protein levels in combination-treated A549 cells (Figure 4C). Furthermore, an increase in p50 nuclear localization was seen from vehicle-treated to Debio 1143-treated cells and Debio 1143-treated cells and combination-treated H2030 cells (Figure 4D). Hence, combination of Debio 1143 and JQ1 induces the non-canonical NF-κB signaling cascade, and canonical NF-κB signaling is repressed to baseline levels following combination treatment in the combination-sensitive H1975 cell line.

### Debio 1143 and JQ1 combination reduces IAP levels and induces ripoptosome formation

**Figure 5:**
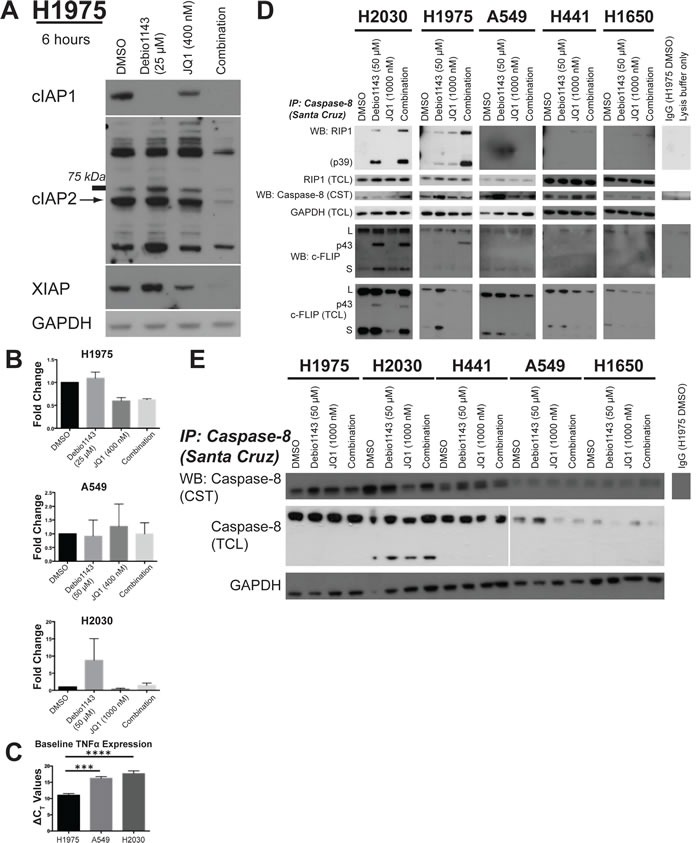
Debio 1143 and JQ1 co-treatment reduces IAP levels and leads to formation of the ripoptosome **A.** Immunoblots of IAP protein levels following 6 hours of treatment with Debio 1143 and/or JQ1. Arrow indicates band of correct molecular weight for cIAP2. **B.** *TNFα* mRNA expression following 6 hour treatment with Debio 1143 and/or JQ1.**C.** Baseline *TNFα* mRNA expression in H1975, A549, and H2030 cells. **D.** Immunoprecipitation of caspase-8 followed by immunoblots of ripoptosome components to measure formation of the ripoptosome following Debio 1143 and/or JQ1 treatment. Cleavage product of RIP1 (p39) indicates active ripoptosome. Cleavage product of c-FLIP (p43) indicative of caspase-8 activity and functional ripoptosome. **E.** Immunoblot of procaspase-8 protein levels.

SMAC mimetics function by inhibiting and promoting destruction of IAPs [12–14]. In H1975, H2030, and A549 cells, Debio 1143 nearly completely ablates cIAP1 (Figure 5A, Figure S5A). JQ1 also moderately reduces cIAP1 levels in H1975, A549, and H2030 cells. Protein levels of cIAP1 are undetectable in the Debio 1143/JQ1 combinations (Figure 5A, S5A). Debio 1143 can also bind cIAP2 and XIAP levels but not to the extent seen for cIAP1 [27]. cIAP2 and XIAP levels were most completely suppressed following combination treatment with Debio 1143 and JQ1 in H1975 cells (Figure 5A). In H2030 cells, XIAP levels were reduced below baseline levels following JQ1 and combination treatment (Figure S5B). The reduction in XIAP levels was surprising since XIAP levels are not usually affected by SMAC mimetics [7, 11]. cIAP2 levels were also reduced following JQ1 treatment, but some rebound in cIAP2 protein levels was seen in combination-treated H2030 cells (Figure S5B). cIAP2 levels were also suppressed in A549 cells following the combination treatment, but there was little impact on XIAP (Figure S5B). Together, these data indicate that suppressing several IAPs is associated with greatest growth inhibition of lung adenocarcinoma cells.

SMAC mimetics can induce expression of TNFα, which activates the NF-κB pathway [12–14, 28]. Furthermore, JQ1 pleiotropically reduces expression of many transcripts including *MYC* and *FOSL1* [29–32]. Debio 1143 enhanced *TNFα* mRNA expression in H2030 cells, but not H1975 or A549 (Figure 5B), and JQ1 did not affect *TNFα* mRNA expression. However, baseline expression of *TNFα* mRNA was significantly higher in H1975 cells than the other two lines (Figure 5C). *FOSL1* transcription was reduced by JQ1 treatment in JQ1-sensitive cell lines, but no further decrease was observed following combination treatment with Debio 1143. Instead, Debio 1143 increased expression of *FOSL1* in all lines tested regardless of sensitivity to the agent (Figure S6A). Consistent with other reports for lung adenocarcinoma, JQ1 had little impact on *MYC* mRNA (Figure S6B) [32]. Overall, we found no correlation between Debio 1143 response, JQ1 response, or combination response and change in expression of the genes tested. We also found no association with response to Debio 1143, JQ1, or the combination and histone H3 lysine-27 acetylation or pan-acetylation of histone H4 (Figure S6C).

TNFα induces formation of the ripoptosome, a cell-death inducing complex that controls both apoptotic and necrotic cell death [16, 17]. Caspase-8 and RIP1 (RIPK1) are ripoptosome components. In Debio 1143-sensitive H2030 and H1975 cells, Debio 1143 alone strongly promotes co-immunoprecipitation of RIP1 and c-FLIP (especially p43) with caspase-8; this is indicative of ripoptosome formation. Further increases in RIP1 and caspase-8 co-immunoprecipitation were seen following combination treatment (Figure 5D). Both the p39 cleavage product of RIP1 and the p43 cleavage product of c-FLIP_L_ co-immunoprecipitated with caspase-8, marking a functional ripoptosome [17, 33]. In contrast, co-immunoprecipitation of caspase-8 with RIP1 was not observed in three Debio 1143-insensitive cell lines - H441, H1650, and A549 (Figure 5D).

Total levels of c-FLIP isoforms (c-FLIP_L_ and c-FLIP_S_) were reduced following JQ1 and combination treatment in four of the lines tested regardless of sensitivity to Debio 1143. In H2030 cells, only JQ1-treated cells showed decreased c-FLIP protein levels, not combination-treated H2030 cells (Figure 5D). However, total cell lysate increases in c-FLIP_p43_ levels were also observed in H2030 cells treated with JQ1 (alone or in combination with Debio 1143) (Figure 5D). Combined, these results indicate a functioning ripoptosome is formed in the two cell lines most sensitive to the combination of Debio 1143 and JQ1. Also, inhibition of ripoptosome formation is removed even in Debio 1143-insensitive lines following JQ1 treatment. Procaspase-8 levels do correlate with greatest response to the combination of Debio 1143/JQ1 combination. Of the five lines tested for ripoptosome formation, the three lines with highest baseline caspase-8 levels were the most sensitive to the combination (Figure 5E). Although H441 is insensitive to Debio 1143 treatment, the combination of Debio 1143 and JQ1 super-additively inhibits growth more than either agent alone (Figure S7). Overall, ripoptosome formation correlates with Debio 1143 sensitivity and JQ1 reduces protein levels of anti-apoptotic c-FLIP. Debio 1143 and JQ1 combination sensitivity are more closely associated with baseline procaspase-8 protein levels than with ripoptosome formation.

### Debio 1143-containing combinations inhibits xenograft growth of lung adenocarcinomas

**Figure 6:**
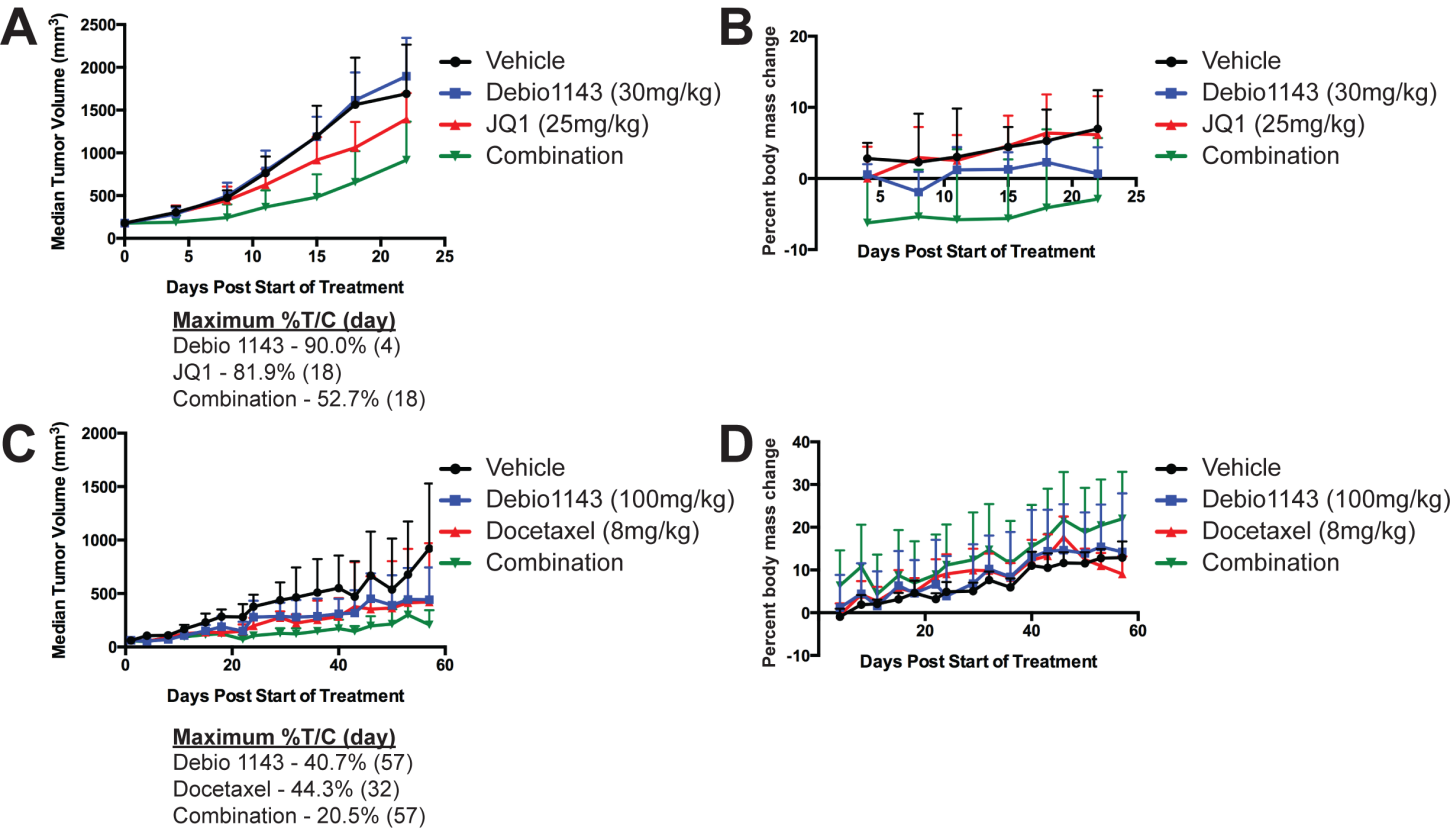
Debio 1143 in combination with either JQ1 or docetaxel delays growth of H1975 and A549 lung adenocarcinoma in xenograft mouse models Median tumor volume of H1975 xenografts **A.** or percent body mass change **B.** following treatment with Debio 1143 (30 mg/kg p.o.) and/or JQ1 (25 mg/kg i.p.). Median tumor volume of A549 xenografts **C.** or percent body mass change **D.** following treatment with Debio 1143 (100 mg/kg p.o.) and/or Docetaxel (8 mg/kg, i.v.). Error bars are for standard deviation. Maximum percent tumor volume/control (%T/C) for **A.** and **C.** also noted for each treatment arm.

Xenografts of H1975 and A549 cells were implanted in nude mice to test the efficacy of Debio 1143 in combination with either JQ1 or docetaxel. Limited effects of the combination of Debio 1143 and JQ1 treatment on tumor volume were observed in H1975 xenografts treated with 30mg/kg Debio 1143; 25mg/kg JQ1 (maximum %treated/control [%T/C] = 52.7%) (Figure 6A). At these drug concentrations, body weights were reduced early in treatment, indicative of toxicity, but body weights stabilized over time (Figure 6B). Pro-inflammatory dose limiting toxicity was observed at higher doses of each agent (data not shown). In contrast, little overt toxicity was observed for the combination of Debio 1143 and docetaxel at 100mg/kg Debio 1143; 8mg/kg docetaxel (Figure 6D), which was highly effective on A549 xenografts (maximum %T/C = 20.5%) (Figure 6C).

## DISCUSSION

Drug combination treatments are essential for more effective cancer therapies. Here, we describe a combination screen of the SMAC mimetic Debio 1143 with 128 other agents against a panel of lung adenocarcinoma cell lines. Sensitivity to Debio 1143 was not associated with any mutational genotype. Debio 1143 in combination with the microtubule stabilizer paclitaxel, topoisomerase inhibitor SN-38, or the bromodomain inhibitor JQ1 synergistically inhibited growth and clonogenicity, and induced apoptosis in lung adenocarcinoma cells. Debio 1143 reduced levels of cIAP1 in all lines tested and variably affected other IAPs. Debio 1143 also modulated NF-κB signaling with increasing nuclear localization of p52 (non-canonical signaling). Debio 1143/JQ1 combination treatment impaired canonical p50 nuclear localization in the most Debio 1143-sensitive cell line tested. Furthermore, the combination of Debio 1143 and JQ1 induced formation of the cell-death inducing ripoptosome complex. Procaspase-8 protein levels most closely correlated with sensitivity to the combination of Debio 1143 and JQ1. Debio 1143 in combination with JQ1 or docetaxel gave modest to robust *in vivo* growth inhibition, respectively, in lung adenocarcinoma xenografts.

Cell cycle and checkpoint inhibitors, such as the pan-cyclin dependent kinase (CDK) inhibitor flavopiridol and polo-like kinase inhibitor 1 (PLK1) BI-2536 among others, were among the most effective combination partners with Debio 1143 in our combination screen. These targets are important in maintaining mutant *KRAS* lung adenocarcinomas [34, 35] suggesting that relieving some cellular anti-apoptosis restraints with SMAC mimetics may be effective in concert with some of these therapies. Tyrosine kinase inhibitors also combined effectively with Debio 1143 in our sensitization screen. Two cell lines with T790M EGFR gatekeeper mutations were especially sensitive to combinations with Debio 1143 and either afatinib, foretinib, or dasatinib. Foretinib can inhibit MET and TAM family receptors (TYRO3, AXL, MERTK) [36]. MET is indicated as a source of resistance to EGFR inhibitor therapy and crosstalk with EGFR can modulate signaling [22, 37]. TAM family receptors are also implicated as resiliency receptors that bypass EGFR-targeted therapy [38]. Dasatinib inhibits Src-family kinases implicated in driving EGFR-independent resistance mechanisms to EGFR inhibitors [37]. Debio 1143 in combination with MEK inhibition also had great effects on *KRAS* mutant cell lines. MEK inhibitor combinations have shown promise for *KRAS* mutant tumors [39].

IAPs are E3-ubiquitin ligases expressed at high levels in cancer [1]. SMAC mimetics induce conformational changes in cellular IAPs leading to auto-ubiquitination and proteasomal degradation [12–14]. Debio 1143 binds the BIR3 domain of cIAP1 and cIAP2 more strongly than XIAP [27]. We found that Debio 1143 reduces cIAP1 levels as a single-agent and in combinations, regardless of the growth sensitivity of cell lines to Debio 1143. Therefore, cIAP1 reduction is not sufficient to promote apoptosis. Debio 1143 variably reduced XIAP and cIAP2, but all three IAPs were suppressed in the most sensitive line, H1975.

Other SMAC mimetics induce non-canonical and canonical NF-κB signaling, which was confirmed as well for Debio 1143 [5, 6, 12]. Loss of the cellular IAPs increases protein levels of NF-κB-inducing kinase (NIK), facilitating the activity of the non-canonical NF-κB signaling pathway [12, 40]. Induction of canonical NF-κB signaling radiosensitizes glioblastoma cells and can differentiate glioblastoma stem-cell-like cells [19, 41]. Furthermore, SMAC mimetic-treated pancreatic adenocarcinoma cells rely on canonical NF-κB signaling to be sensitized to gemcitabine [42]. Activation of the canonical NF-κB pathway by SMAC mimetics leads to increased *TNFα* mRNA expression [12–14, 28]. The SMAC mimetic sensitive cell lines had strong baseline *TNFα* expression (H1975), or *TNFα* was strongly induced in another (H2030). Increased activity of these pathways cannot fully explain the pro-apoptotic effects of Debio 1143 because these pathways are active in Debio 1143-insensitive cells. Therefore, other mechanisms are behind the activity of Debio 1143 in this panel of lung adenocarcinoma cell lines tested.

Beyond increased NF-κB activity, SMAC mimetic treatment and IAP depletion induces ripoptosome formation [16, 17]. Depletion of IAPs causes a TNFα-independent accumulation of RIP1 within the cytosol; this accumulation of RIP1 serves as a scaffold for ripoptosome formation [16, 17]. In chronic lymphocytic leukemia cells, the inability to form ripoptosomes is linked to cell line resistance to SMAC mimetic treatment [43]. Furthermore, in acute lymphoblastic leukemia, ripoptosome components, such as RIP1, are required to induce cell death [44]. We found that Debio 1143 induces ripoptosome formation in Debio 1143-sensitive cell lines. The degree to which the cells form ripoptosomes following Debio 1143 treatment did not fully correlate with response to Debio 1143. H2030 is less sensitive to Debio 1143 than H1975, but H2030 had greater induction or ripoptosome assembly than H1975 following Debio 1143 treatment. This suggests that the degree of ripoptosome assembly is less important than just forming the ripoptosome complex in order to induce cell death following SMAC mimetic treatment.

SMAC mimetics are excellent partner agents for combination therapies because they antagonize anti-apoptotic proteins. Our combination sensitization screen uncovered several agents that super-additively combined with Debio 1143. Many of these combinations were confirmed to be synergistic and apoptosis-inducing, underscoring the notion that antagonizing IAPs results in chemosensitization.

Bromodomain inhibitors greatly enhance the effects of Debio 1143. Bromodomain inhibition leads to broad transcriptional and epigenetic changes with complex biological outcomes [29–32, 45]. *KRAS*-driven lung adenocarcinomas with wild-type *LKB1* are more sensitive to bromodomain inhibitor JQ1 than those with alterations in *LKB1* [31]. This pattern was observed in the three *KRAS* mutant cell lines tested in our panel. H441 has functional LKB1 and is sensitive to JQ1 treatment, whereas H2030 and A549 (non-functional LKB1) were less sensitive to JQ1 treatment. Furthermore, JQ1 sensitivity does not seem to correlate with *MYC* mRNA reduction, a common mechanism seen in other cancers [29, 30, 32]. Reduction in *FOSL1* expression may be more likely facilitating the effects of bromodomain inhibition in these lung adenocarcinoma cells [32].

The closest association between Debio 1143 and JQ1 combination sensitivity and biological response was with components of the ripoptosome. Ripoptosome formation correlated with response to Debio 1143 as a single-agent, but not in combination as H441 cells were sensitive to the combination, but no ripoptosome formation was observed following combination treatment. Also, loss of both c-FLIP_L_ and c-FLIP_S_ ripoptosome components did not correlate with response to the combination. This is somewhat surprising given that ripoptosome formation is inhibited by c-FLIP_L_ and promoted by c-FLIP_S_, however, this is cell context specific [17, 46]. The p39 cleavage product of RIPK1 and p43 cleavage product of c-FLIP_L_ are indicative of caspase-8 activity [16, 33, 47]. Caspase-8 is the caspase responsible for facilitating ripoptosome-mediated cell death [16, 17]. The most striking correlation we were able to see with Debio 1143 and JQ1 combination sensitivity was with baseline protein levels of procaspase-8. Procaspase-8 protein levels are overexpressed in lung cancers [48]. JQ1 may be contributing to the effects of the combination mechanism by depleting c-FLIP levels. Depletion of c-FLIP has differential effects on lung adenocarcinoma cell lines, but caspase-8 is necessary for lines to be sensitive to c-FLIP depletion [49]. By modulating these proteins it is possible to enhance sensitivity to SMAC mimetics.

We describe here the potential utility of Debio 1143 in combination with taxanes, topoisomerase inhibitors, or bromodomain inhibitors. The combination of Debio 1143 with both paclitaxel and carboplatin is in clinical trials for lung squamous cell carcinoma, platinum-refractory ovarian carcinoma, and triple negative breast cancer (NCT01930292). Other drug classes that combined effectively with Debio 1143 in our screen targeted cell cycle and checkpoint kinases, epigenetic modifiers, tyrosine kinases, and the NF-κB pathway. Hence the pro-apoptotic activities of Debio 1143 may enhance response to a broad range of therapeutic targets.

*In vivo* xenograft testing of some combinations confirmed modest activity of the combination of Debio 1443 and JQ1 and marked activity of the Debio 1143 and docetaxel combination for lung adenocarcinoma. With the Debio 1143 and JQ1 combination, there was some pro-inflammatory dose limiting toxicity observed. Inclusion of anti-inflammatory agents may ameliorate some of these effects. We also observed enhanced activity in A549 xenografts following Debio 1143 plus docetaxel treatment relative to the single agents. This is noteworthy as A549 cells were insensitive to this combination in our *in vitro* screen. SMAC mimetics have been reported to be more effective in vivo for treatment of pancreatic adenocarcinoma cell [28]. It is possible that systemic treatment of the whole mouse induces microenvironmental changes or alters paracrine signaling which further sensitizes the cells to combination treatment.

We have used sensitization screening to identify combination compound regiments with Debio 1143 to inhibit growth and induce apoptosis of lung adenocarcinoma cells. These combinations are excellent candidates for further therapeutic development as they include agents already in clinical use (taxanes, topoisomerase and MEK inhibitors, etc.) or novel agents with attractive targets and target interactions (bromodomain inhibitors). Our findings provide a rationale for the combination of Debio 1143 with paclitaxel in an ongoing clinical trial in several cancer types (ClinicalTrials.gov Identifier: NCT01930292). The especially strong impact of the combination of bromodomain inhibitors and SMAC mimetics warrants further investigation to identify the most important transcriptional targets, and the mechanisms that foster cooperation to induce cancer cell apoptosis.

## MATERIALS AND METHODS

### Cell culture and antibodies

Lung adenocarcinoma cell lines were cultured as described previously [22]. H1975, H2030, A549, H1650, and H441 were obtained from the American Type Culture Collection (ATCC). H2228 cells were a gift from J. Peter Koo (Yale). PC9, PC9/BRC1, H3255, and H3255 XLR were a gift from Katerina Politi (Yale). PC9/BRC1 and H3255 XLR cells are EGFR inhibitor resistant cells and have the EGFR T790M gatekeeper mutation [23].

Antibodies used include: PARP, phospho-p70S6K, p70S6K, phospho-AKT^Ser473^, AKT, phospho-ERK, ERK, β-actin, caspase-8 (mouse), p105/p50, and p100/p52 (Cell Signaling); RIP1 (BD Pharmingen); caspase-8 (goat), glyceraldehyde-3-phosphate dehydrogenase (GAPDH) (Santa Cruz); histone H3 (Abcam); c-FLIP (Enzo Life Sciences).

### Combinatorial compound screening (Figure 1A)

Six cell lines were seeded at 1,000 cells per well in 16 μL into 384-well plates using a MultiDrop Combi Reagent dispenser (Thermo). Plates were centrifuged at 1000 × *g* to evenly disperse cells and cells allowed to adhere overnight. The following day, the cells were incubated with 20 nL each of 128 compounds using a PlateMate Plus automated pipetting system (MatrixTechCorp, Thermo). The 128 compounds were arrayed on two 384-well master plates with sixty-four agents in a five-point, eight-fold dilution series. The highest concentration was usually 10 μM of compound.

After addition of the agents from the compound master plates, 4 μL of either 1% DMSO (vehicle control) or Debio 1143 diluted in medium at 5X the GI_10_, GI_25_, or GI_50_ concentrations was added. The concentrations of Debio 1143 were tailored according to experimentally determined GI values for each cell line. A MultiDrop Combi reagent dispenser (Thermo) distributed Debio 1143 or vehicle to each well. 20% DMSO was used as a complete growth inhibition control. Cells were treated in duplicate. 10 μL of CellTiterGlo reagent was added to each well after seventy-two hours of incubation and luminescence was measured. All plates had Z’-scores of 0.5 or higher except for two plates with scores of 0.42 and 0.47.

### Growth assays

Cells were plated at 1,500-4,000 cells in 100 μL per well into 96-well black-bottom plates and allowed to adhere over night. The next day, cells were treated with respective compounds. Seventy-two hours later, 60 μL CellTiterGlo reagent was added to each well and luminescence was measured to determine percent growth inhibition (%GI).

Vehicle control was highest concentration of DMSO used in treatment conditions, between 0.1-0.3% (v/v) DMSO. Complete growth inhibition signal control was 20% (v/v) DMSO.

### Statistical analyses

AUC measurements were performed as described previously [24]. Combination index (CI) values were calculated using CompuSyn software (ComboSyn) for the non-automated Chou-Talalay experiments [25].

### Colony formation assays

400 cells were plated in six-well plates. Cells were treated the next day and then again three days later for a total of six days of incubation. Compounds were washed off the cells and allowed to grow five more days before being stained with 0.01% crystal violet.

### Flow cytometry

250,000 cells were plated in each well of a six-well tissue culture plate. After cells adhered overnight, they were treated with compound or DMSO vehicle for 72 hours. Next, adherent and non-adherent cells were collected, resuspended in annexin V binding buffer, and stained with both propidium iodide and annexin V-APC according to the manufacturer's protocol (BD Biosciences, San Jose, CA). Cells were analyzed using an LSRII flow cytometer.

### Cell fractionation

Cells were incubated with compounds for 24 hours. Cells were washed twice with 1X Dulbecco's PBS (without magnesium or calcium) and then collected by scraping in a third wash of PBS. The cell solution was divided in half. Cells in both portions were collected by centrifugation. One of the pellets was lysed in 1% SDS buffer (40 mM Tris HCl, pH 7.4; 150 mM NaCl, 1% SDS, 10% glycerol), incubated at 100°C for 10 minutes, sonicated for 5 minutes (alternating pulses 15 seconds on, 15 seconds off) and cleared by centrifugation. The remaining cell pellet was washed once with RSB (reticulocyte standard buffer [10 mM NaCl, 1.5 mM CaCl_2_, 10 mM Tris HCl, pH 7.4]) and re-centrifuged. The wash RSB was removed and the cells were then swelled on ice for 10 minutes in 10-20 volumes of RSB. The swollen cells were disrupted with a motorized Teflon pestle. The nuclei were pelleted at 1,000 × *g* for 5 minutes at 4°C. The supernatant (cytosolic fraction) was removed. The nuclear fraction was washed three times more with RSB and resuspended in 1% SDS buffer. Nuclear fractions were incubated at 100°C for 10 minutes, sonicated, and cleared as described for whole cell extracts. Cytosolic fractions were cleared three times more by centrifugation, with the supernatant transferred after each clearing step. SDS and glycerol were added to a final concentration of 1% and 10%, respectively, and cytosolic fractions were incubated at 100°C for 10 minutes. Comparable cell equivalent portions of nuclear and cytosolic fractions were analyzed by gel electrophoresis.

### RNA isolation and real-time PCR

RNA was isolated and prepared for real-time PCR as described previously [22]. Fold changes were calculated using the 2^−ΔΔCt^ method normalized to GAPDH transcript levels.

### Co-immunoprecipitation

Cells were incubated for six hours with compounds. Cells were washed twice with 1X Dulbecco's PBS (without magnesium or calcium) and then lysed in immunoprecipitation lysis buffer (20 mM Tris-HCl, pH 7.4; 150 mM NaCl; 1% Triton X-100; 10% glycerol; 0.5 mM dithiothreitol; Roche Complete protease inhibitor cocktail). Approximately 500 μg of protein was immunoprecipitated with 2 μg of caspase-8 (Santa Cruz) antibody or goat IgG (Santa Cruz) and 20 μL of protein A/G agarose beads (Thermo) with rotation at 4°C for two hours. Beads were washed four times with the lysis buffer. 45 μL of 2X Laemmli sample buffer was then added to the beads and the samples were incubated at 100°C for 10 minutes.

### Western blotting

Samples were resolved by gel electrophoresis and transferred to PVDF membrane. After blocking with 5% (w/v) milk in 1X TBS-T (0.1% Tween-20), cells were incubated overnight with indicated primary antibodies. Wash buffers were chosen according to the manufacturers’ recommendations for each antibody. Appropriate species-specific horseradish peroxidase-conjugated secondary antibodies (Thermo) were added prior to chemiluminescence detection (Thermo West Pico or Femto).

### Xenografts

Appropriate approval from the local institutions Animal Care and Use Committee was obtained and guidelines followed. H1975 (2×10^6^) or A549 (5×10^6^) cells, in 100 μL Matrigel, were implanted subcutaneously in the right flanks of 6-8 or 4-6 week old, female Balb/c nude mice (Shanghai Laboratory Animal Co. LTD, Shanghai, China), respectively. Animals were randomized to groups of 8 animals with mean group tumor volumes of 150-200 mm^3^ and the treatments started the same day. Debio 1143 (Debiopharm International SA) was administered by oral gavage 5 days a week for 3 weeks either at 100 mg/kg (A549) or 30 mg/kg (H1975). Docetaxel (NorthCarolina Chemlabs) was administrated intravenously once a week for 3 weeks (8 mg/kg). JQ1 (Abmole Bioscience Co., Limited, Hong Kong) was formulated in 10% hydroxypropyl-β-cyclodextrin (Sigma-Aldrich, #C0926) and administered by intraperitoneal injection 5 days a week for 3 weeks at 25 mg/kg. Tumor volume and mouse weight was monitored every 3-4 days. Tumor volume measured with the formula *V* = *l* × *w* × *w*/*2*.

## SUPPLEMENTARY MATERIAL FIGURES AND TABLES




