# Supplementary material for: SMAC mimetic Debio 1143 synergizes with taxanes, topoisomerase inhibitors and bromodomain inhibitors to impede growth of lung adenocarcinoma cells
Source: Oncotarget. 2015 Oct 16;6(35):37410–25. doi: 10.18632/oncotarget.6138 (PMC4741938; doi:10.18632/oncotarget.6138)
Supplement: Supplementary file 1 [file oncotarget-06-37410-s001.pdf]

SMAC mimetic Debio 1143 synergizes with taxanes, topoisomerase inhibitors and bromodomain inhibitors to impede growth of lung adenocarcinoma cells

Supplementary Material

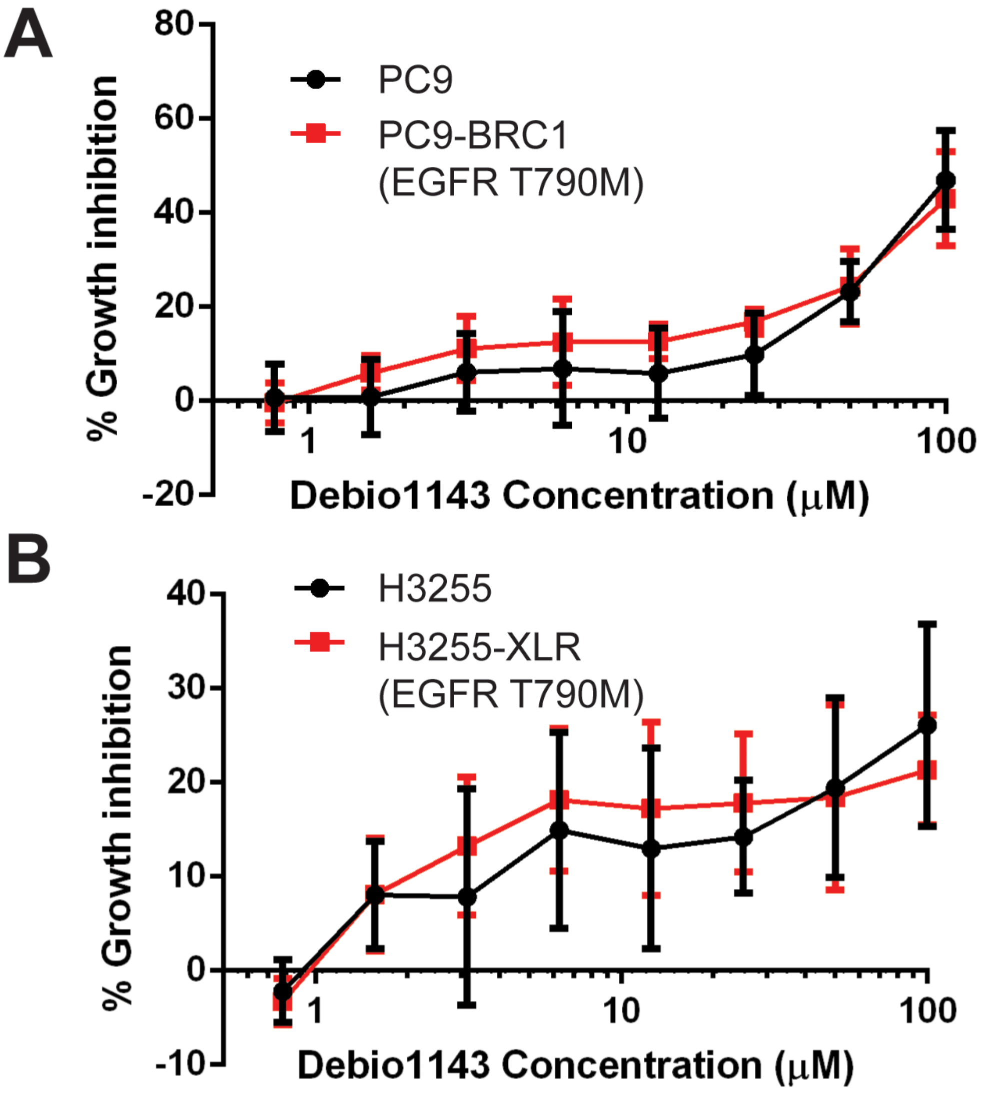

Figure S1. EGFR T790M mutation does not define sensitivity to Debio 1143. (A) Growth inhibition response curves of isogenic PC9 and PC9/BRC1 T790M cell lines following treatment with Debio 1143. (B) Growth inhibition response curves of isogenic H3255 and H3255-XLR T790M cell lines following treatment with Debio 1143.

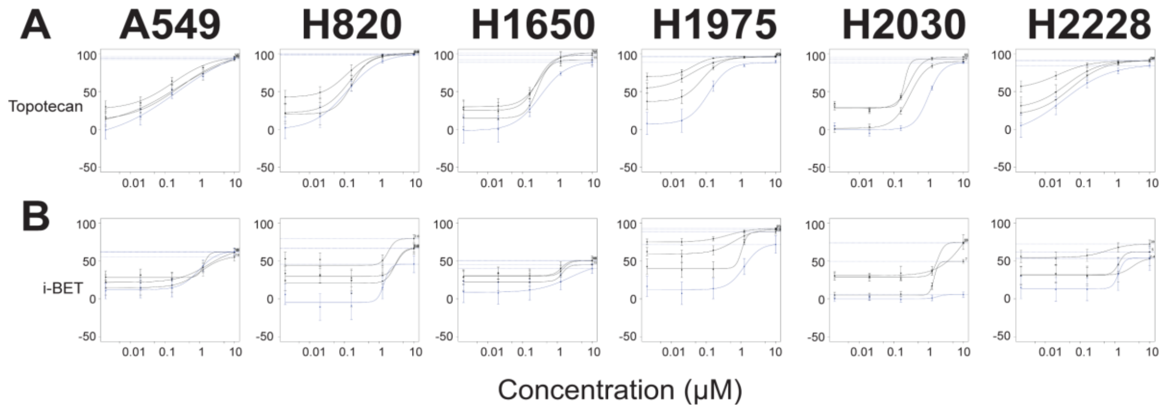

Figure S2. Debio 1143 and either topotecan (A) or iBET (B) combination mimics effects of Debio 1143 and either SN-38 or JQ1, respectively, in lung adenocarcinoma cell lines.

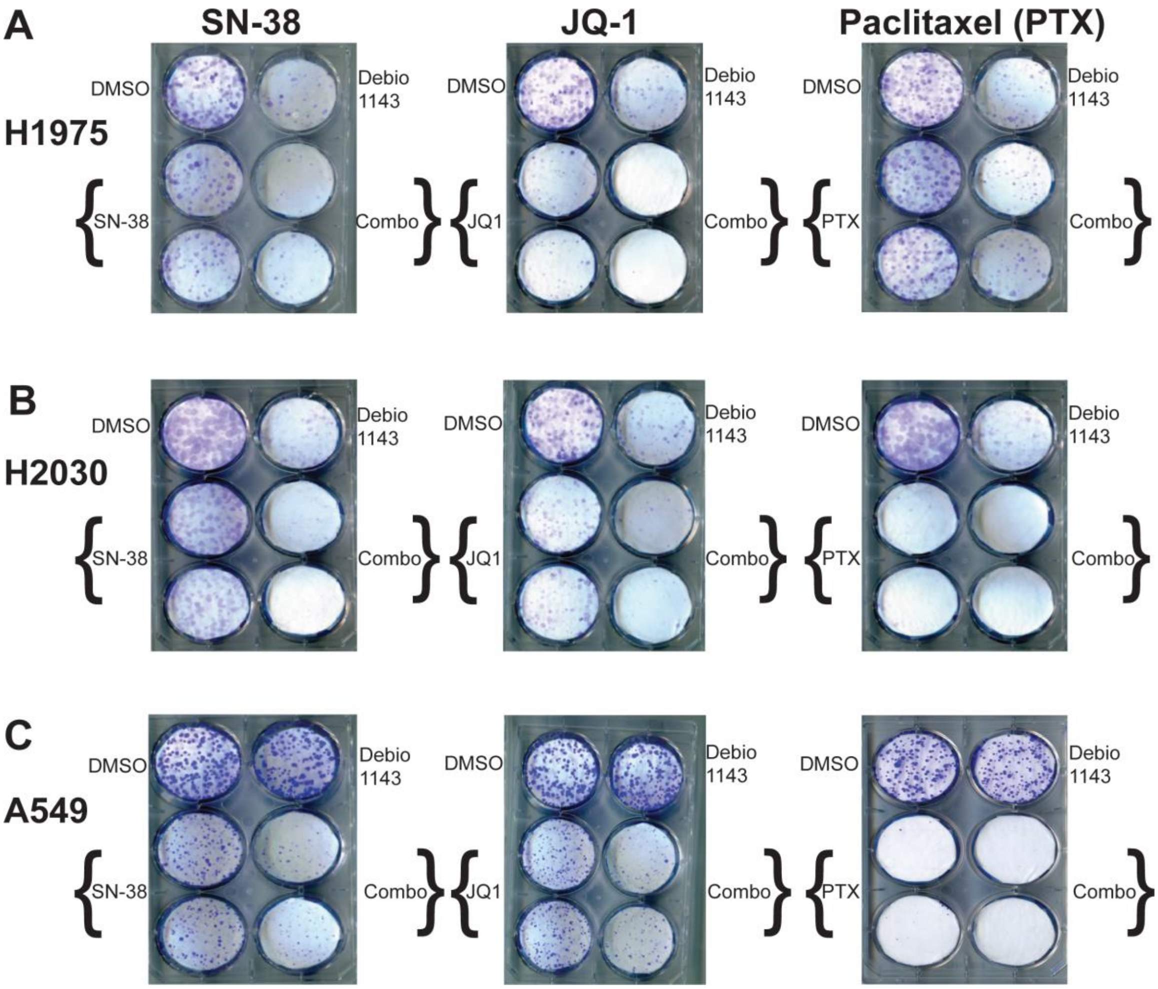

Figure S3. Debio 1143 combinations inhibit clonogenicity of lung adenocarcinoma cells. Colony formation assays in H1975 (A), H2030 (B), and H2030 (C) cells with Debio 1143 in combination with SN-38, JQ1, or paclitaxel.

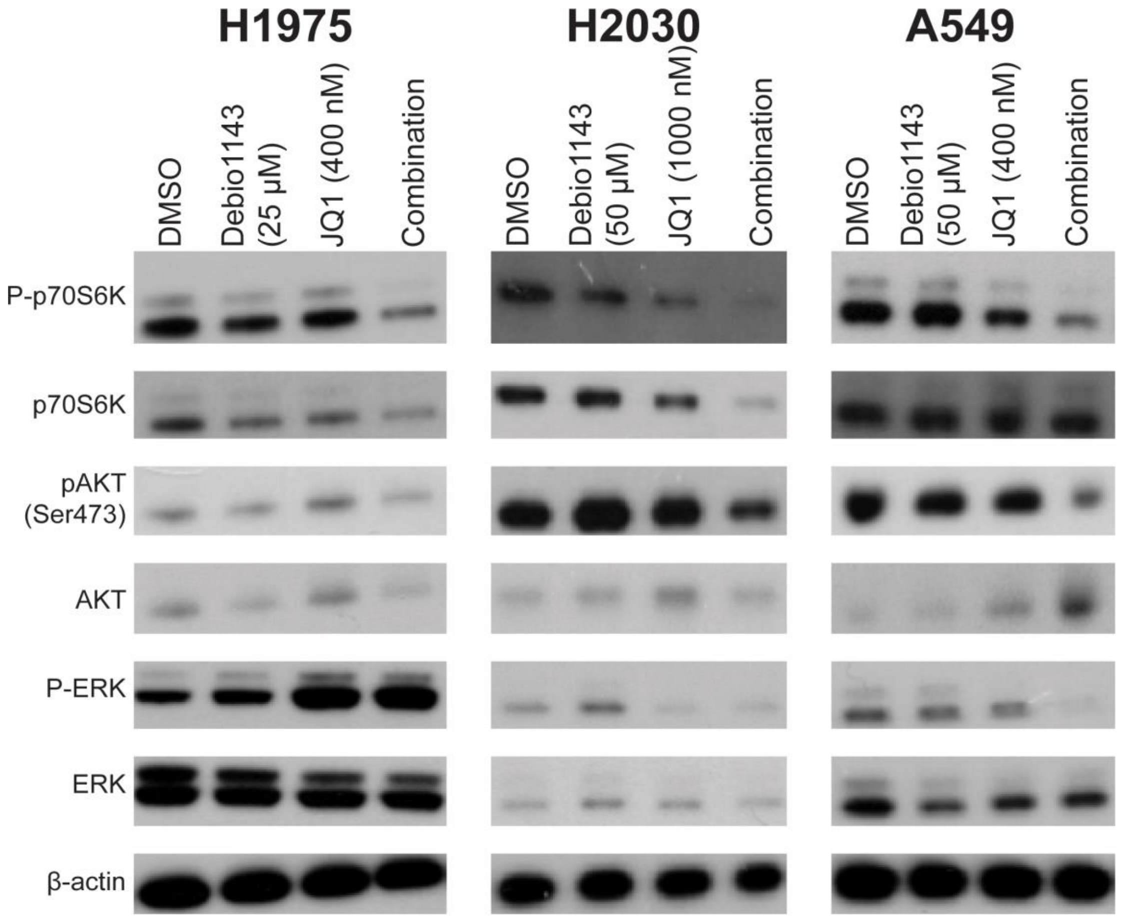

Figure S4. Modulation of PI3K/AKT pathway and MAPK pathway signaling following 24 hours of treatment with Debio 1143, JQ1, or the combination.

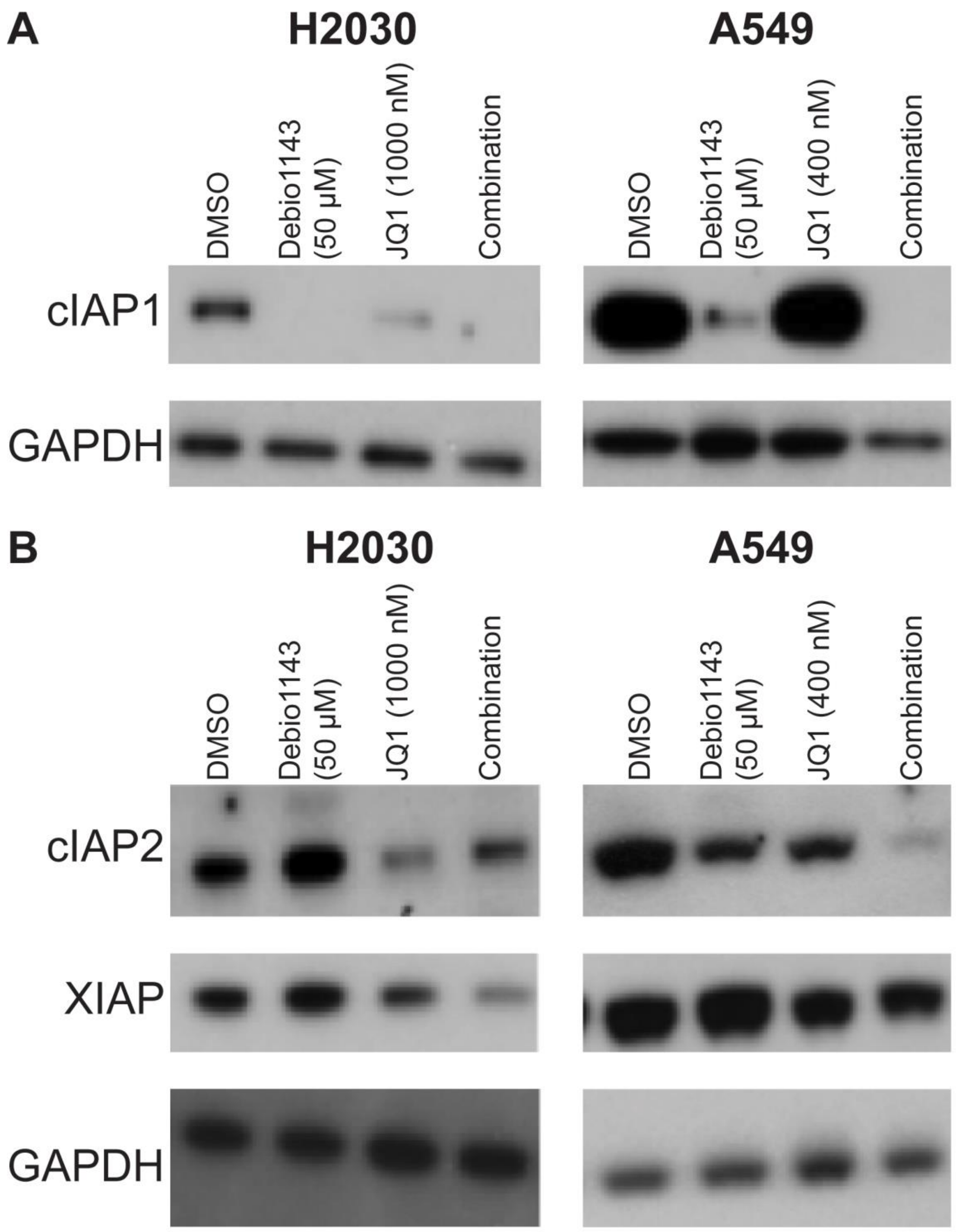

Figure S5. Effect of Debio 1143, JQ1, and the combination on protein levels of cIAP1, cIAP2, and XIAP in H2030 and A549 cells. Immunoblots of cIAP1 (A) or XIAP or cIAP2 (B) in H2030 and A549 cells following 24 hours of treatment with Debio 1143 and/or JQ1.

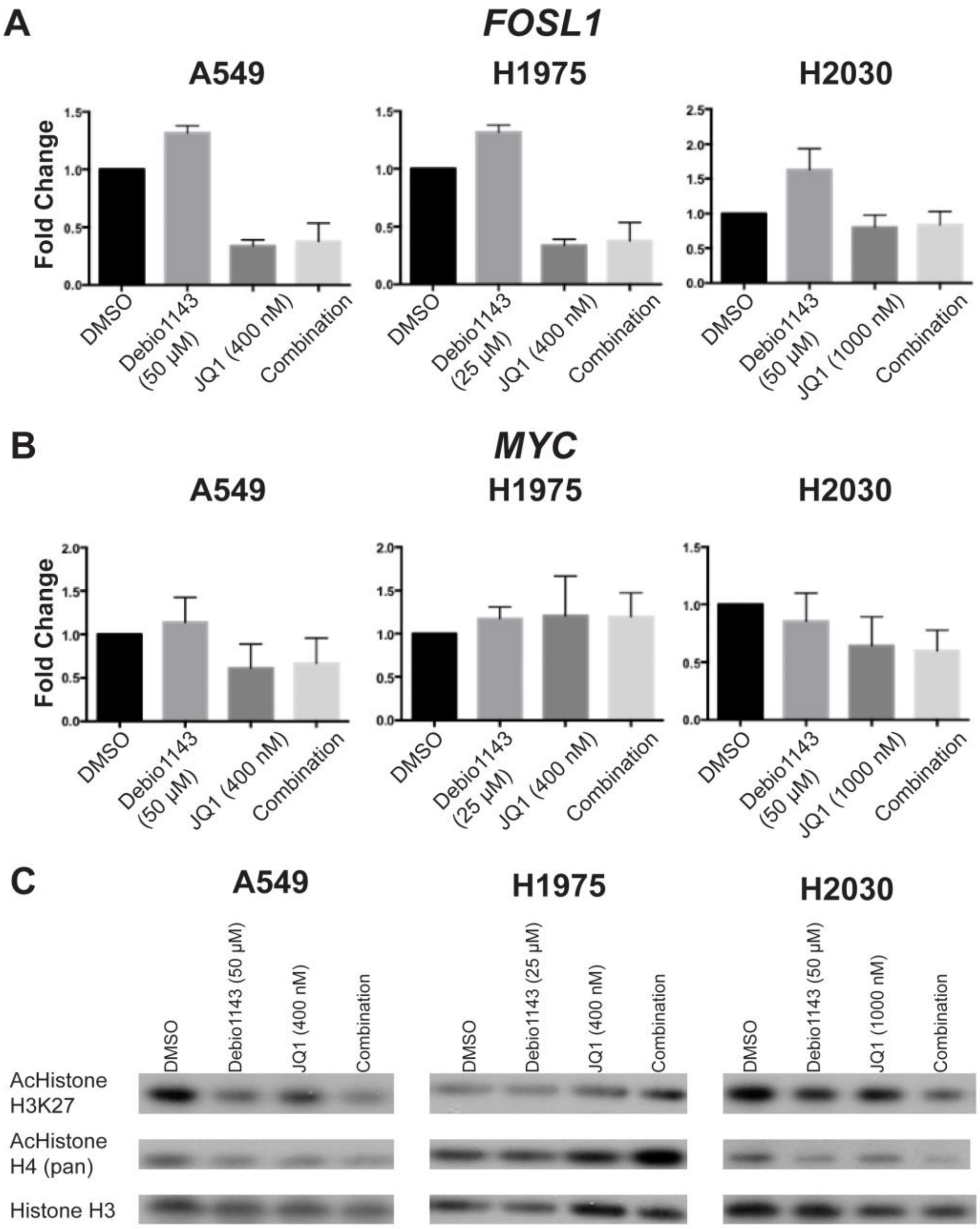

Figure S6. mRNA expression of JQ1 targets in lung adenocarcinoma cells and histone acetylation levels following Debio 1143, JQ1, and combination treatment. (A) *FOSL1* mRNA expression levels following 6 hours of treatment with Debio 1143, JQ1, or the combination. (B) *MYC* mRNA expression levels following 6 hours of treatment with Debio 1143, JQ1, or the combination. (C) Immunoblots of nuclear protein extracts

indicating levels of acetylated histone H3 lysine 27 or pan-acetylated histone H4 following 6 hr of Debio 1143, JQ1, or combination treatment.

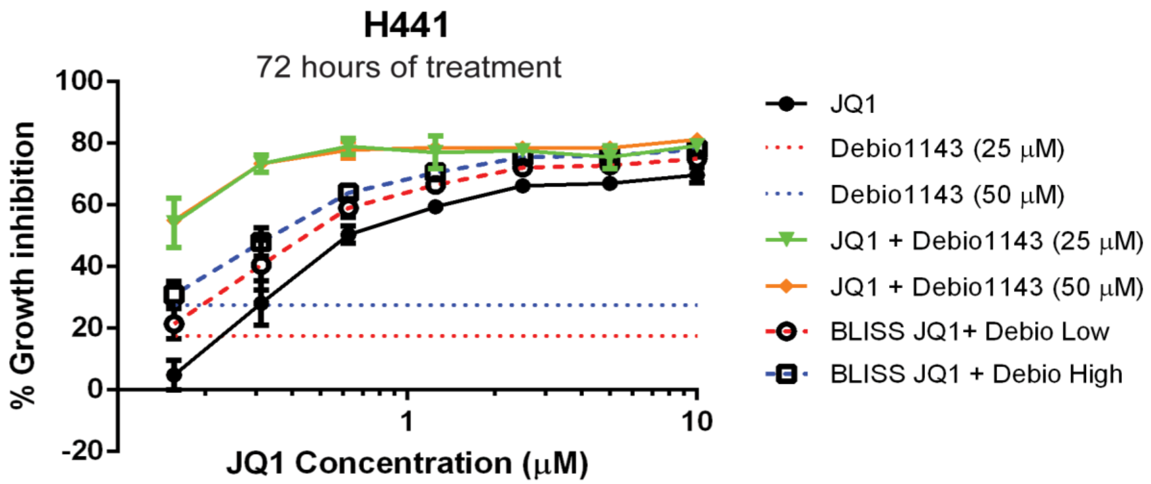

Figure S7. The combination of Debio 1143 and JQ1 super-additively inhibits the growth of H441 cells. The dotted line indicates the effect of Debio 1143 alone. The black line is the JQ1 growth response curve. The dashed line indicates the predicted effect of the combination based upon the Bliss independence model. The green and orange curves are the actual effects of the combination.
