# Supplementary material for: SMAC mimetic Debio 1143 synergizes with taxanes, topoisomerase inhibitors and bromodomain inhibitors to impede growth of lung adenocarcinoma cells
Source: Oncotarget. 2015 Oct 16;6(35):37410–25. doi: 10.18632/oncotarget.6138 (PMC4741938; doi:10.18632/oncotarget.6138)
Supplement: Supplementary file 2 [file oncotarget-06-37410-s002.pdf]

	A549_Debio1143	H1650_Debio1143	H1975_Debio1143
17-DMAG	82	9	33
2-deoxy-D-glucose	106	87	75
5-fluorouracil	31	73	41
5-nonyloxytryptamine oxalate	54	69	69
ABT-263	48	16	31
ABT-888	125	128	118
AR-A014418	107	104	122
Atorvastatin	61	63	52
AZD-4547	113	119	127
AZD-7762	9	12	45
AZD-8931	17	40	89
Bay-11-7082	73	60	51
BEZ-235	8	84	87
BI-2536	10	29	5
BI-D1870	45	50	49
BIBW-2992	4	25	40
BMS-754807	3	74	46
Bortezomib	16	19	34
Bosutinib	96	52	113
BRD-7389	22	72	56
BX 513	97	106	119
Carboplatin	95	116	90
Carfilzomib	87	21	27
Cerulenin	100	90	117
CH-5424802	72	36	54
CIP 13-74	2	75	82
CMPD-1	46	118	106
Crizotinib	114	123	65
Curcumin	120	101	68
CYT-387	110	113	84
Dactinomycin	83	8	37
Dapagliflozin	80	100	80
Dasatinib	55	14	28
Decitabine	119	68	103
Digoxin	86	18	35
Docetaxel	69	4	22
Dovitinib	64	94	70
Efavirenz	111	107	99
EHT-1864	77	77	86
Embelin	112	126	121
Enzastaurin	126	114	124
Erlotinib	36	48	58
ES 936	104	81	116
Etoposide	15	89	47
Flavopiridol	1	17	43

	A549_Debio1143	H1650_Debio1143	H1975_Debio1143
Foretinib	68	20	16
GANT61	90	103	74
GDC 0449	75	83	83
GDC-0941	47	22	21
Gemcitabine	40	5	7
GGTI-298	76	55	61
GSK1120212	53	30	29
GSK690693	51	38	63
HBX-41108	79	45	18
Homoharringtonine	42	6	9
Hydroxychloroquine sulfate	121	127	126
I-BET	89	47	23
Imatinib	123	124	120
IMD 0354	70	3	36
JK 184	23	2	4
JNK inhibitor II	99	82	100
JQ1	52	57	15
KP372-1	59	15	10
Lapatinib	44	61	76
Metformin	74	98	81
Mitomycin C	11	46	125
MK-1775	65	23	17
MK-2206	98	109	91
ML 130	62	93	66
MLN-4924	28	26	72
MLN-8237	60	41	3
MS-275	13	70	1
Nelfinavir	105	115	115
NH125	58	31	25
Nutlin-3	41	95	78
NVP-231	88	110	97
NVP-LDE225	92	111	107
NVP-TAE684	5	34	24
Obatoclax	6	44	92
Oligomycin	85	53	11
Orlistat	57	86	67
OSI-906	35	51	59
Oxaliplatin	94	125	110
Paclitaxel	29	1	14
PD-0332991	27	71	95
PD173074	115	120	123
Pemetrexed	20	79	20
PF 429242	49	88	73
PF 431396	33	37	26
PF-573228	93	97	101

	A549_Debio1143	H1650_Debio1143	H1975_Debio1143
PF3644022	38	58	88
PHA 665752	108	85	109
Piperlongumine	109	78	105
PLX 4032	91	96	112
PNU 74654	116	108	111
PX 12	128	117	98
Rapamycin	25	28	48
Ritonavir	122	121	104
RO4299097	124	99	55
Rosiglitazone	118	112	114
SB 218078	12	54	13
SB225002	34	39	39
Simvastatin	67	91	44
SN-38	71	7	6
Sorafenib	102	105	108
STA-4783	21	32	128
Stattic	101	66	71
Sunitinib	117	122	96
Syk Inhibitor	127	102	102
Temsirolimus	7	65	94
Thioridazine	66	80	79
Tipifarnib	81	62	42
Tivantinib	19	42	50
Topotecan	56	11	2
Tozasertib	14	49	38
Triapine	18	35	32
Trifluoperazine	39	67	57
Trifluorothymidine	50	43	53
Triptolide	32	10	19
UNC0638	37	76	62
Vandetanib	24	24	85
Verapamil	63	64	93
Vinorelbine	30	13	8
Vorinostat	26	59	60
WZ4002	43	27	12
XAV 939	103	56	64
YM155	78	33	30
Zibotentan	84	92	77

	H2030_Debio1143	H2228_Debio1143	H820_Debio1143
17-DMAG	22	48	9
2-deoxy-D-glucose	103	80	66
5-fluorouracil	88	43	41
5-nonyloxytryptamine oxalate	79	76	49
ABT-263	54	26	11
ABT-888	127	128	128
AR-A014418	80	117	97
Atorvastatin	64	61	55
AZD-4547	125	121	125
AZD-7762	52	23	35
AZD-8931	82	102	47
Bay-11-7082	97	74	46
BEZ-235	60	47	78
BI-2536	7	11	15
BI-D1870	43	44	76
BIBW-2992	78	67	8
BMS-754807	95	45	64
Bortezomib	25	3	24
Bosutinib	50	111	112
BRD-7389	89	39	48
BX 513	74	100	98
Carboplatin	117	89	100
Carfilzomib	30	14	42
Cerulenin	106	94	91
CH-5424802	29	32	77
CIP 13-74	48	88	105
CMPD-1	58	103	127
Crizotinib	116	79	123
Curcumin	85	84	96
CYT-387	123	125	111
Dactinomycin	2	7	27
Dapagliflozin	112	82	71
Dasatinib	28	15	17
Decitabine	124	107	93
Digoxin	27	30	33
Docetaxel	1	4	2
Dovitinib	91	93	73
Efavirenz	118	95	94
EHT-1864	67	58	74
Embelin	120	126	119
Enzastaurin	126	115	88
Erlotinib	61	51	53
ES 936	102	112	63
Etoposide	68	50	54
Flavopiridol	13	31	3

	H2030_Debio1143	H2228_Debio1143	H820_Debio1143
Foretinib	42	24	13
GANT61	109	90	87
GDC 0449	96	85	51
GDC-0941	31	27	10
Gemcitabine	38	17	19
GGTI-298	92	65	50
GSK1120212	18	35	67
GSK690693	84	63	62
HBX-41108	45	42	26
Homoharringtonine	16	33	7
Hydroxychloroquine sulfate	108	122	118
I-BET	23	46	84
Imatinib	115	119	116
IMD 0354	11	21	21
JK 184	9	9	18
JNK inhibitor II	49	105	89
JQ1	6	22	38
KP372-1	8	13	4
Lapatinib	83	92	37
Metformin	113	86	82
Mitomycin C	12	6	60
MK-1775	40	19	25
MK-2206	81	71	126
ML 130	98	62	86
MLN-4924	51	55	32
MLN-8237	59	16	12
MS-275	35	18	31
Nelfinavir	121	113	113
NH125	19	37	34
Nutlin-3	99	83	72
NVP-231	119	96	83
NVP-LDE225	111	108	107
NVP-TAE684	72	12	44
Obatoclax	21	57	40
Oligomycin	46	49	59
Orlistat	94	69	70
OSI-906	71	66	61
Oxaliplatin	128	118	121
Paclitaxel	3	2	6
PD-0332991	100	68	52
PD173074	107	110	117
Pemetrexed	104	29	101
PF 429242	70	78	39
PF 431396	62	36	23
PF-573228	114	91	109

	H2030_Debio1143	H2228_Debio1143	H820_Debio1143
PF3644022	65	60	90
PHA 665752	93	87	106
Piperlongumine	122	104	79
PLX 4032	66	101	115
PNU 74654	57	106	108
PX 12	90	116	120
Rapamycin	17	25	85
Ritonavir	110	127	114
RO4299097	86	52	80
Rosiglitazone	101	114	122
SB 218078	36	64	36
SB225002	53	28	22
Simvastatin	33	81	103
SN-38	5	10	16
Sorafenib	44	120	99
STA-4783	39	123	20
Stattic	105	99	56
Sunitinib	87	109	110
Syk Inhibitor	77	124	104
Temsirolimus	26	41	102
Thioridazine	69	77	68
Tipifarnib	47	97	124
Tivantinib	63	40	45
Topotecan	4	8	14
Tozasertib	56	38	28
Triapine	32	34	69
Trifluoperazine	41	70	57
Trifluorothymidine	24	53	81
Triptolide	10	1	1
UNC0638	37	59	43
Vandetanib	34	56	30
Verapamil	75	73	58
Vinorelbine	15	20	5
Vorinostat	14	54	92
WZ4002	55	75	65
XAV 939	20	98	75
YM155	73	5	29
Zibotentan	76	72	95
